# The reliability and validity of rehabilitation set of the international classification of functioning, disability, and health in assessing Chinese tumor patients

**DOI:** 10.1371/journal.pone.0349504

**Published:** 2026-06-03

**Authors:** Qianqian Sun, Zhi Zou, Wei Guo, Rui Li, Suchen Zhao, Hao Yu, Xiaoguang Yang, Zeyang Yu, Luwen Zhang, Hongyu Zhai, Tiebin Yan

**Affiliations:** 1 Xiangyang Central Hospital, Affiliated Hospital of Hubei University of Arts and Science, Xiangyang, China; 2 Guangzhou Sport University, Guangzhou, China; 3 School of Health Services Management, Southern Medical University, Guangdong, China; 4 Department of Rehabilitation Medicine, Sun Yat-sen Memorial Hospital, Sun Yat-sen University, Guangzhou, China; Chinese University of Hong Kong, HONG KONG

## Abstract

**Background and aim:**

There is currently no comprehensive scale suitable for assessing the rehabilitation progress of Chinese tumor patients. This study aimed to evaluate the psychometric properties of the Rehabilitation Set of the International Classification of Functioning, Disability, and Health for that purpose.

**Methods:**

The Chinese version of the International Classification of Functioning, Disability, and Health’s Rehabilitation Set (ICF-RS) was used by trained health professionals to assess 1055 Chinese tumor patients. The internal consistency of the responses was assessed in terms of Cronbach’s alpha coefficient. Construct validity was explored through factor analysis. Test-retest reliability, inter-rater reliability, and criterion validity were evaluated using a subset of 121 patients. Criterion validity was quantified by correlating the scores with those generated using the Hospital Anxiety and Depression scale (HADS), the Eastern Cooperative Oncology Group (ECOG) performance status evaluation and the modified Barthel Index (MBI).

**Results:**

The Cronbach’s alpha for the rehabilitation set’s scores was 0.87, indicating good internal consistency. The test-retest reliability analysis showed that most categories had an intraclass correlation coefficient in excess of 0.75, suggesting good reliability when the rehabilitation set is applied with tumor patients. Inter-rater reliability also demonstrated intraclass correlation greater than 0.75 for most categories. Construct validity was supported by the finding that 18 categories had factor loadings exceeding 0.40. After removing categories with lower factor loadings, the cumulative variance explained increased to 44.6% for the Activity and Participation component and 57.3% for the Body Functions component. Criterion validity was supported by significant associations with established instruments. Specifically, 66.7% (20/30) of the ICF-RS categories correlated significantly with the MBI, with effect sizes (r) ranging from 0.19 to 0.52 (p < 0.05). In contrast, only 10% (3/30) of categories demonstrated significant correlations with the ECOG ratings (ρ = 0.21–0.22, p < 0.05). Furthermore, categories related to emotional functions and psychological demands showed significant correlations with the HADS, with moderate effect sizes (ρ = 0.37–0.41, p < 0.001).

**Conclusions:**

The Chinese version of the ICF-RS exhibits promising psychometric properties in tumor patients, providing preliminary evidence for its clinical application. It demonstrates good internal consistency, high test-retest reliability and reliable inter-rater consistency. The moderate correlation with MBI ratings and HADS scale scores, along with the distinct factors identified through factor analysis, further supports the instrument’s validity and utility when applied to this population. The data confirm that the ICF-RS is a reliable tool for assessing tumor patients’ functioning and disability. However, the construct validity results recommend further work before the instrument is widely used with tumor patients.

## Introduction

Tumors are a significant health challenge worldwide and the second leading cause of death according to the World Health Organization (WHO), affecting around 192 million people worldwide [1]. In 2022, China reported approximately 4,824,700 new cases with different tumors and 2,574,200 tumor-related deaths [2]. Beyond mortality, tumor patients face substantial daily challenges during and after treatment, including physical fatigue, pain, and psychological stress [3–7]. The disease experience is inherently stressful, often leading to significant emotional distress, fear, anxiety, and depression, which may be further exacerbated by treatment side effects [8]. Furthermore, social stigma and discrimination can negatively impact quality of life and social participation, potentially resulting in isolation [9,10]. These multifaceted challenges underscore the need for comprehensive, patient-centered assessment of functioning that extends beyond clinical outcomes to encompass the full spectrum of rehabilitation needs.

Despite these multifaceted challenges, clinical attention has historically prioritized treatment outcomes, often neglecting comprehensive assessment of patients’ social functioning. This oversight is particularly evident in functional assessment, where existing tools typically capture only limited aspects of a patient’s experience [11, 12]. For instance, the Hospital Anxiety and Depression Scale (HADS) assesses emotional state [10], the Eastern Cooperative Oncology Group’s (ECOG) performance status scale evaluates physical condition [13], and the modified Barthel Index (MBI) measures ability in activities of daily living [14,15]. This limitation makes it difficult for healthcare professionals to identify specific barriers to participation in family and social activities and to inform personalized, holistic rehabilitation plans.

To address this need, the World Health Organization published the International Classification of Functioning, Disability and Health (ICF) in 2001, providing a comprehensive framework for assessing individual functioning and disability across body functions, activities and participation, and environmental factors [16–19]. The ICF is now widely recognized as a useful assessment tool for comprehensively evaluating health, functioning and disability in the context of environmental and individual factors [20–22]. However, its full version contains 1,495 categories, rendering it impractical for routine clinical use. In response to this limitation, the ICF Research Center developed the ICF Rehabilitation Set (ICF-RS), comprising 30 secondary core categories: 9 for body functions and 21 for activities and participation [23] (S1 Table). The ICF-RS offers a comprehensive evaluation of a patient’s status, considering body functions, daily activities, social involvement, and environmental and cultural contexts. It assists in evaluating rehabilitation effectiveness, monitoring quality, and guiding improvements, while also identifying potential barriers and informing adjustments to treatment plans [24]. As a universal functional assessment tool, the ICF-RS is suitable for various healthcare rehabilitation settings, promoting recovery and enhancing quality of life.

Substantial psychometric evidence supports the reliability and validity of the ICF-RS in diverse clinical populations. Polish validation studies demonstrated very high intraclass correlation coefficients (ICC values ranging from 0.793 to 1.000), indicating excellent reproducibility and consistency [25]. In Chinese rehabilitation populations, the ICF-RS-17, a simplified 17-item version of the original set, demonstrated strong internal reliability (latent class reliability coefficient = 0.963), with the estimated functional competence strongly correlated with the Barthel Index (r = 0.81) [26]. The Brief ICF Core Set for Chronic Obstructive Pulmonary Disease (COPD) showed excellent content validity (Scale-level Content Validity Index = 0.943) and adequate construct validity [27]. Additionally, ICF-based instruments developed for specific cancer populations—including the Brief Core Set Questionnaire for Breast Cancer (BCSQ-BC) [28] and the Cancer Functional Assessment Set (cFAS) [29]—have demonstrated good to excellent psychometric properties in measuring comprehensive functioning. These validation studies confirm that ICF-based tools can reliably capture multidimensional aspects of patient functioning and are responsive to changes in rehabilitation status.

The Chinese version of the ICF-RS is now widely applied in clinical practice in China, supported by a corresponding mobile application [30,31] that facilitates convenient assessments for clinicians and patients [32]. This tool has demonstrated good reliability and validity in multi-center Chinese rehabilitation research. While the psychometric properties of the ICF-RS have been demonstrated for neurological, musculoskeletal, and cardiopulmonary disorders [33], evidence regarding its use with tumor patients is lacking. Given that tumor populations present unique challenges distinct from other chronic conditions—including cancer-related fatigue [34], treatment-induced peripheral neuropathy [35], and psychosocial impacts of cancer diagnosis and survival [36]—dedicated validation in this population is warranted. Therefore, this study aimed to assess the reliability and validity of the Chinese version of the ICF-RS in Chinese tumor patients.

## Methods

### Participants

A total of 1,055 tumor patients admitted to Xiangyang Central Hospital in China’s Hubei Province from October 1, 2023 to November 30, 2024 were enrolled. To ensure clinical homogeneity and a uniform functional baseline, several strategic criteria were applied. We enrolled patients who did not require surgery and were receiving their first course of radiotherapy, chemotherapy, or targeted therapy; all participants had a hospital stay of 7 days. Inclusion criteria were: (1) diagnosed with one or more tumors at the early clinical stage (TNM Stages I and II); (2) aged 18 years and over; and (3) willingness to give informed consent. Exclusion criteria were: (1) critically ill with unstable vital signs; (2) unwilling to cooperate throughout the evaluation process; and/or (3) severe anxiety and/or depression (HADS score: 15–21 points). Demographic and clinical data were collected for each patient. The rigorous selection process focused on clinical parsimony by minimizing the confounding effects of advanced-stage complications, such as cachexia or end-of-life symptoms, as well as acute psychiatric crises. The cohort included patients with various types of malignancies, including lung, breast, and gastrointestinal cancers. However, by restricting the study to early-stage diseases and first-course treatments, a population with comparable rehabilitation potential and disease severity was ensured. This provided a stable foundation for the psychometric evaluation of the ICF-RS.

The study was approved by the Ethics Review Board of Xiangyang Central Hospital (IRB No.2023−098) and registered on China’s Clinical Trials Registry (ChiCTR2300079108). Written informed consent was obtained from all the participants, in agreement with the declaration of Helsinki. Prior to enrollment, potential participants were provided with a detailed Participant Information Sheet. The consent process was conducted by trained study staff, who verbally informed each patient that participation was entirely voluntary. It was explicitly stated, both verbally and within the written informed consent form, that declining to participate or withdrawing from the study at any time would have no impact on their clinical care, treatment, or access to hospital services. Written informed consent was obtained from all participants before data collection commenced.

### Assessments

Assessments were conducted using the ICF-RS mobile application. Based on previous research indicating that sample sizes above 80–100 cases yield minimal additional improvements [37] in agreement metrics for the intraclass correlation coefficient (ICC), and considering methodological recommendations for reliability studies suggesting a sample size of 1–4 times the number of items [38], a sub-sample of 121 participants was selected from the recruited patients for inter-rater and test-retest reliability assessments to ensure precision and rigor. The selection of 121 cases is further supported by a recent psychometric validation study [39], which demonstrated that carefully defined clinical samples can provide robust internal consistency and high test-retest reliability when rigorous protocols are applied. The selection was performed via simple random sampling without replacement using the “Select Cases” function in SPSS 26.0 (IBM Corp., Armonk, NY, USA), ensuring that every participant had an equal probability of selection and eliminating subjective selection bias. To ensure representativeness, demographic characteristics and baseline scores of this sub-sample were compared with the full cohort, showing no significant differences (*p* > 0.05). Assessments were performed at two time points by two licensed therapists, each with at least three years of clinical experience, who had completed intensive face-to-face ICF-RS training and passed a certification assessment, ensuring accurate and consistent application.

### Test-retest reliability

A longitudinal design was implemented with a follow-up assessment conducted after a 3-day interval. The initial assessment was conducted within 24 hours of admission, with patients instructed to respond based on their functioning, activity, and participation during the previous two weeks. The 3-day interval minimized bias from significant changes in participant condition. The same assessor completed both assessments.

### Inter-rater reliability

For inter-rater reliability, the two trained assessors independently conducted assessments without consultation or discussion regarding participant scores, ensuring ratings were based solely on individual judgment. Ratings from each rater were collected and recorded separately for accurate comparison.

### Criterion validity

Criterion validity was tested by correlating ICF-RS scores with established reference measures: the ECOG performance status scale for physical functioning, the HADS for emotions, and the MBI for functional ability in daily activities. Each participant completed the ICF-RS alongside these reference instruments within the same session to ensure temporal consistency.

The ECOG physical performance score is a widely used clinical tool in oncology for assessing patients’ functional and performance status, serving as a key indicator for treatment decisions, prognosis assessment, and eligibility screening in clinical trials [40,41]. This tool effectively reflects the impact of disease on overall patient functioning, with its core domains (e.g., activity endurance and self-care ability) closely aligned with the “Activities and Participation” domain of the ICF; consequently, evaluating the correlation between the ICF-RS and ECOG provides a direct test of the ICF-RS’s validity in capturing cancer-related functional impairments [40,41].

Complementing functional assessment, the Hospital Anxiety and Depression Scale (HADS)—specifically developed for hospital settings—acts as a brief, easy-to-administer instrument that effectively screens for anxiety and depression in cancer patients [42], making it widely used for psychological health assessment in oncology populations [11]. In this study, total subscale scores are interpreted as: 0–7 (normal), 8–14 (borderline abnormal), and 15–21 (abnormal) [12]. To ensure the focus on general rehabilitation status and minimize the confounding impact of acute psychiatric crises, patients scoring in the severe range (15–21) were excluded from the study. Within the ICF framework, anxiety and depression are categorized under “Emotion Function s” in the “Body Functions” domain, and as psychological distress constitutes a central component of cancer patients’ health experience (with significant impacts on quality of life and treatment adherence), using HADS as an external criterion enables focused evaluation of the ICF-RS’s validity in measuring patients’ mental health functioning [11,42].

The Modified Barthel Index (MBI), primarily employed to assess patients’ activities of daily living and reflect functional independence levels, has demonstrated good reliability, validity, and internal consistency in cancer populations—additionally, it is sensitive to changes in functional status and closely associated with survival and prognosis [43]. Since one of the core domains of the ICF-RS is the assessment of “Activities” and “Participation”, and the MBI specifically measures the most fundamental and central components of the “Activities” domain, a high level of criterion-related validity between the ICF-RS and MBI would provide direct evidence that the ICF-RS accurately reflects patients’ basic functional status [43,44].

### Assessment tools

The ICF responses were gathered using the Chinese ICF-RS mobile application. Each of the 9 Body Functions categories and 21 Activities and Participation categories was rated using appropriate qualifiers (S2 Table) that indicate the extent to which impairment, limitations and restrictions of functioning were self-perceived. The rating scales offer 7 levels: no/ mild/ moderate/ severe/ complete impairment or difficulty plus not specified and not applicable. “Not specified” or “not applicable” responses were treated as a missing value in the analyses.

The ECOG performance status scale has six grades ranging from 0 to 5. The grades are defined as follows: 0, fully active; 1, restricted in strenuous activity but ambulatory and able to do light work; 2, ambulatory and capable of all self-care but unable to work; 3, capable of only limited self-care; 4, completely disabled; and 5, dead [45]. The HADS comprises two subscales: HADS-A for detecting anxiety and HADS-D for detecting depression. Each subscale with seven categories rated on a 4-point scale (scores 0–21 per subscale), with higher scores indicating greater anxiety or depression [46]. The MBI consists of ten categories, with a maximum score of 100 indicating normal ability; scores of 71–99, 46–70, 21–45, and ≤21 indicate mild, moderate, heavy, and complete dependence, respectively [47].

### Data analysis

Internal consistency was assessed using Cronbach’s alpha, with a value of ≥0.70 considered acceptable [33,48]. We also examined whether removing any category would improve each subscale’s reliability. Test-retest reliability was evaluated using intraclass correlation coefficients (ICCs) based on a two-way mixed-effects model, absolute agreement, single rater type [ICC(3,1)]. Inter-rater reliability was assessed using two-way random-effects model, absolute agreement, single rater type [ICC(2,1)], ICC values were interpreted as follows: < 0.50, poor; 0.50–0.75, moderate; > 0.75, excellent [49,50]. Inter-rater reliability was evaluated using inter-rater ICCs with kappa values categorized as <0.50, poor; 0.50–0.75, moderate; > 0.75, excellent; and 0.9–1, almost perfect [50,51].

Construct validity was examined using the Kaiser–Meyer–Olkin (KMO) measure and exploratory factor analysis, rather than Structural Equation Modeling (SEM), as the goal was to assess sampling adequacy and factorability rather than test a predefined model [52,53]. The KMO measure evaluated whether variable correlations justified factor analysis, a necessary step before confirmatory modeling. Given the exploratory nature of this study and sample size limitations, KMO and factor analysis were deemed more appropriate. The KMO measure was first applied to assess sampling adequacy, followed by Bartlett’s test of sphericity to examine inter-category correlations. Principal component analysis with varimax rotation was then performed, retaining factors with eigenvalues >1. Categories with factor loadings below 0.40 were excluded to ensure meaningful factor contributions [33,54].

Criterion validity was evaluated by correlating ICF-RS scores with three external measures using Spearman’s rank correlation coefficients. To control for Type I errors in multiple comparisons, the Bonferroni correction was applied to adjust the p-values. The resulting correlation strengths were interpreted as weak (0.10–0.29), moderate (0.30–0.49), or strong (≥0.50) [55].

## Results

### Patient demographics

The participants were 59.1% male and 40.9% female with an average age of 62.4 (±10.1) years. Table 1 presents average scores on the assessment scales, while Table 2 illustrates the distribution of tumor cases classified by system. The ceiling and floor effects of the ICF-RS categories were evaluated to assess the scale’s ability to distinguish between different levels of functioning. The ceiling effect (percentage of patients scoring 0) ranged from 0.14% to 92.32%, while the floor effect (percentage scoring 4) ranged from 0.00% to 46.47% (S3 Table). Based on a 15% threshold, significant ceiling effects were present in the majority of Activities and Participation categories, most notably in *d530* Toileting (92.32%) and *d540* Dressing (92.12%). Conversely, a significant floor effect was identified only for *b640* Sexual functions (46.47%). These findings reflect the relatively high functional status and low disability burden characteristic of early-stage tumor patients during the initial rehabilitation phase.

**Table 1:** Demographic characteristics of the cancer patients.

Patients	Full sample n = 1055	Sub-sample n = 121
**Sex, n(%)**		
Male	623(59.1)	61(50.4)
Female	432(40.9)	60(49.6)
**Age,** Mean (SD)	62.4 ± 10.1	61.7 ± 10.0
**Scores**		
ECOG	1.9 ± 0.5	
HADS	10.5 ± 6.6	
HADS-A	4.5 ± 3.6	
HADS-D	6.0 ± 3.8	
MBI	95.3 ± 11.0	

Note: All of the scores in the table are medians; the values in parentheses are means ± the standard deviation (SD). ECOG: Eastern Cooperative Oncology Group instrument; HADS: Hospital Anxiety and Depression scale; MBI: modified Barthel Index. The entire sample was used to assess internal consistency, construct validity and criterion validity, while the sub-sample was employed to evaluate inter-rater reliability and test-retest reliability.

**Table 2:** Distribution of the tumor cases by system.

Tumor site	Number of cases	Percentage
Respiratory System	421	39.9%
Digestive System	283	26.8%
Reproductive System	198	18.8%
Hematopoietic System	72	6.8%
Urinary System	43	4.1%
Other	38	3.6%
Total	1055	100.0%

Note: The participants in this study presented with a diverse range of tumor types. The most prevalent malignancy was lung cancer, followed by gynecological cancers, gastrointestinal tumors and breast cancer. The tumor stages were classified according to the TNM staging system and clinical staging (Stages I–IV) and all the patients were diagnosed with Stage I to II.

### Internal consistency

Internal consistency analysis yielded a Cronbach’s α of 0.87 for the total scale, demonstrating excellent reliability across the 30 categories (Table 3). This result demonstrates good internal consistency among the items, indicating that they reliably measure the multidimensional construct of functioning as conceptualized by the ICF framework. Furthermore, the Cronbach’s α if item deleted values for individual categories ranged from 0.87 to 0.89. Because the deletion of any single category did not result in a substantial increase in the overall α coefficient, it can be concluded that all categories contribute meaningfully to the internal consistency of the ICF-RS. This level of internal reliability supports the use of the scale as a stable tool for assessing functional status in this population.

**Table 3:** Internal consistency and Cronbach’s α if item deleted (n = 1055).

Component and category		Cronbach’s α
b130	Energy and drive functions	0.87
b134	Sleep functions	0.87
b152	Emotional functions	0.87
b280	Sensation of pain	0.87
b455	Exercise tolerance functions	0.87
b620	Urination functions	0.89
b640	Sexual functions	0.87
b710	Mobility of joint functions	0.87
b730	Muscle power functions	0.87
d240	Handling stress and other psychological demands	0.87
d410	Changing basic body position	0.87
d415	Maintaining a body position	0.87
d420	Transferring oneself	0.87
d450	Walking	0.87
d455	Moving around	0.86
d465	Moving around using equipment	0.87
d510	Washing oneself	0.87
d520	Caring for body parts	0.87
d530	Toileting	0.87
d540	Dressing	0.87
d550	Eating	0.87
d570	Looking after one’s health	0.87
d640	Doing housework	0.87
d230	Carrying out daily routine	0.87
d470	Using transportation	0.89
d660	Assisting others	0.87
d710	Basic interpersonal interactions	0.87
d770	Intimate relationships	0.87
d850	Remunerative employment	0.88
d920	Recreation and leisure	0.87
**Overall functioning**		**0.87**

### Test-retest reliability

In the first part of the study, all 121 participants completed the scale on two separate occasions with a three-day interval. The resulting ICCs ranged from 0.73 to 1, all of which were statistically significant (*p* ≤ 0.001), indicating a high level of stability and consistency over time (Table 4). Furthermore, the narrow 95% confidence intervals observed for each ICC further support the scale’s good test-retest reliability.

**Table 4:** Test-retest and inter-rater reliability results (n = 121).

Component and Category			Test-retest			Inter-rater		
			ICC	95% interval	*p*	ICC	95% interval	*p*
**Body functions**	b130	Energy and drive functions	0.77	0.67-0.85	≤≤0.001	0.90	0.83-0.94	≤0.001
	b134	Sleep functions	0.95	0.93-0.97	≤≤0.001	0.90	0.83-0.94	≤0.001
	b152	Emotional functions	0.99	0.98-0.99	≤0.001	0.88	0.81-0.93	≤0.001
	b280	Sensation	0.92	0.89-0.95	≤0.001	0.98	0.97-0.99	≤0.001
	b620	Urination functions	0.99	0.98-0.99	≤0.001	0.85	0.75-0.91	≤0.001
	b640	Sexual functions	0.98	0.97-0.98	≤0.001	0.94	0.89-0.97	≤0.001
	b455	Exercise tolerance functions	0.97	0.96-0.98	≤0.001	0.73	0.55-0.83	≤0.001
	b710	Mobility of joint functions	1.00	–	≤0.001	0.62	0.37-0.77	≤0.001
	b730	Muscle power functions	0.97	0.96-0.98	≤0.001	0.81	0.69-0.89	≤0.001
**Activity** **and Participation**	d410	Changing basic body position	0.89	0.85-0.93	≤0.001	0.85	0.75-0.91	≤0.001
	d415	Maintaining a body position	0.87	0.80-0.91	≤0.001	0.80	0.67-0.88	≤0.001
	d420	Transferring oneself	1.00	–	≤0.001	0.86	0.77-0.92	≤0.001
	d450	Walking	1.00	–	≤0.001	0.83	0.72-0.90	≤0.001
	d465	Moving around using equipment	0.99	0.98-0.99	≤0.001	0.93	0.89-0.96	≤0.001
	d455	Moving around	0.96	0.95-0.97	≤0.001	0.87	0.79-0.92	≤0.001
	d510	Washing oneself	0.96	0.94-0.97	≤0.001	0.98	0.96-0.99	≤0.001
	d520	Caring for body parts	1.00	–	≤0.001	1.00	–	≤0.001
	d530	Toileting	0.99	0.98-0.99	≤0.001	0.98	0.96-0.99	≤0.001
	d540	Dressing	0.98	0.97-0.99	≤0.001	0.80	0.67-0.88	≤0.001
	d550	Eating	0.98	0.98-0.99	≤0.001	0.90	0.83-0.94	≤0.001
	d640	Doing housework	0.99	0.99-0.99	≤0.001	0.90	0.83-0.94	≤0.001
	d570	Looking after one’s health	0.98	0.97-0.99	≤0.001	0.84	0.73-0.90	≤0.001
	d240	Handling stress and other psychological demands	1.00	–	≤0.001	0.86	0.76-0.91	≤0.001
	d230	Carrying out daily routine	0.95	0.93-0.97	≤0.001	0.83	0.72-0.90	≤0.001
	d770	Intimate relationships	1.00	1.00-1.00	≤0.001	0.68	0.46-0.81	≤0.001
	d470	Using transportation	0.99	0.98-0.99	≤0.001	0.91	0.86-0.95	≤0.001
	d660	Assisting others	0.99	0.98-0.99	≤0.001	0.94	0.89-0.96	≤0.001
	d710	Basic interpersonal interactions	0.97	0.95-0.98	≤0.001	0.66	0.44-0.79	≤0.001
	d850	Remunerative employment	0.99	0.98-0.99	≤0.001	0.87	0.78-0.92	≤0.001
	d920	Recreation and leisure	0.99	0.99-0.99	≤0.001	0.92	0.86-0.95	≤0.001

### Inter-rater reliability

The inter-rater reliability of the ICF-RS scales was evaluated by two independent raters using a sub-sample of 121 participants. The resulting ICC values across categories ranged from 0.62 to 1.0 (95% CI: 0.37–1.0), reflecting excellent overall reliability. Specifically, while most categories achieved ICC values above 0.8, four categories showed slightly lower agreement: *b710* Mobility of Joint Functions (ICC = 0.62) and *b455* Exercise Tolerance Functions (ICC = 0.73), *d770* Intimate relationships (ICC = 0.68), and *d710* Basic interpersonal interactions (ICC = 0.66). Except for a few categories, nearly all categories achieved ICC values above 0.8 (Table 4). This high level of agreement indicates consistent interpretation of scale categories between raters, supporting the robustness of the ICF-RS for use in clinical and research settings. Furthermore, qualitative feedback from the raters suggested that clear category definitions and well-structured response formats also contributed to the assessment tool’s reliability.

### Construct validity

The factor analysis revealed consistent loadings across the two identified dimensions. In the Body Functions dimension, five of the nine categories had factor loadings above 0.41, while two categories (*b710* and *b730*) loaded instead onto the Activity and Participation dimension. Within the Activity and Participation dimension, 13 of the 21 categories exhibited significant factor loadings, ranging from 0.41 to 0.90; however, four categories (*d465*, *d455*, *d640*, and *d710*) were found to load onto the Body Functions dimension. Additionally, three categories (*d570*, *d230*, and *d470*) displayed cross-loadings in both dimensions (S5 Table).

To ensure the structural clarity and statistical parsimony of the tool within a tumor-specific context, categories with factor loadings below 0.40 or those exhibiting significant cross-loadings were removed [56]. Following this refinement, the Body Functions dimension retained 4 categories, and the Activity and Participation dimension retained 13 categories. The decision to remove categories with factor loadings below 0.40, rather than retaining cross-loading items, was made to prioritize the statistical parsimony and structural clarity of the ICF-RS in a tumor-specific context. By refining the scale to include only items with high factor-to-dimension specificity, we significantly improved the cumulative variance explained (from 43.29% to 57.26% for Body Functions) and ensured that each dimension represents a distinct, non-overlapping construct. This approach enhances the tool’s clinical interpretability, although it results in a more streamlined version of the original 30-item set Table 5.

**Table 5:** Construct validity results (n = 1055).

Component and category			Factor loading coefficient		Common factor variance
			Body functions	Activity and Participation	
**Body functions**	b134	Sleep functions	**0.62**	0.03	0.38
	b280	Sensation	**0.62**	0.19	0.42
	b620	Urination functions	**0.59**	0.03	0.35
	b640	Sexual functions	**0.49**	0.04	0.24
	b455	Exercise tolerance functions	**0.44**	0.35	0.31
**Activity** **and Participation**	d410	Changing basic body position	0.27	**0.69**	0.55
	d415	Maintaining a body position	0.24	**0.70**	0.54
	d420	Transferring oneself	−0.01	**0.86**	0.75
	d450	Walking	0.03	**0.89**	0.78
	d510	Washing oneself	0.12	**0.77**	0.60
	d520	Caring for body parts	0.03	**0.88**	0.78
	d530	Toileting	0.02	**0.91**	0.82
	d540	Dressing	−0.02	**0.89**	0.80
	d550	Eating	0.14	**0.72**	0.54
	d570	Looking after one’s health	0.34	**0.69**	0.60
	d230	Carrying out daily routine	0.31	**0.66**	0.53
	d470	Using transportation	0.31	**0.66**	0.53
	d660	Assisting others	0.22	**0.44**	0.24
Eigenvalue (after rotation) Variance explained (after rotation) Cumulative variance explained (after rotation) KMO value Bartlett’s sphericity test value Degrees of freedom *p* value			7.58	2.16	
			44.60%	12.70%	
			44.60%	57.26%	
			0.93		
			12837		
			136		
			≤0.001		

KMO: Kaiser-Meyer-Olkin Measure of Sampling Adequacy.

### Criterion validity

The criterion validity of the ICF-RS scale was evaluated by correlating its scores with those from the HADS, ECOG, and MBI instruments. Spearman correlation analysis indicated significant negative correlations between 20 ICF-RS categories and MBI scores, encompassing 5 categories from the Body Functions dimension and 15 from the Activity and Participation dimension. These inverse relationships—where higher ICF-RS scores correspond to lower MBI scores—support the validity of the ICF-RS in capturing constructs related to activities of daily living (Table 6).

**Table 6:** Correlation of ICF-RS ratings with scores using other instruments (n = 1055).

ICF-RS category		MBI		ECOG		HADS	
		ρ	*p*	ρ	*p*	ρ	*p*
**Body functions**							
b130	Energy and drive functions	0.08	0.373	−0.05	0.581	0.17	0.065
b134	Sleep functions	−0.19	0.037	0.08	0.382	0.04	0.676
b152	Emotional functions	0.10	0.261	0.10	0.275	0.41	≤0.001
b280	Sensation	−0.22	0.016	0.09	0.354	0.20	0.028
b620	Urination functions	−0.32	≤0.001	0.02	0.821	−0.04	0.675
b640	Sexual functions	−0.19	0.056	0.01	0.895	−0.03	0.738
b455	Exercise tolerance functions	−0.15	0.101	0.01	0.882	0.01	0.923
b710	Mobility of joint functions	−0.36	≤0.001	0.21	0.022	−0.04	0.669
b730	Muscle power functions	−0.22	0.017	0.03	0.713	0.18	0.053
**Activity and Participation**							
d410	Changing basic body position	−0.41	≤0.001	0.09	0.322	0.02	0.829
d415	Maintaining a body position	−0.43	≤0.001	0.02	0.802	0.04	0.677
d420	Transferring oneself	−0.11	0.222	0.06	0.512	0.23	0.012
d450	Walking	−0.33	≤0.001	0.08	0.411	0.20	0.031
d465	Moving around using equipment	−0.33	≤0.001	0.22	0.015	0.16	0.080
d455	Moving around	−0.34	≤0.001	0.05	0.600	0.05	0.619
d510	Washing oneself	−0.46	≤0.001	0.16	0.073	0.08	0.388
d520	Caring for body parts	−0.49	≤0.001	0.14	0.114	0.09	0.339
d530	Toileting	−0.42	≤0.001	0.15	0.103	0.09	0.352
d540	Dressing	−0.36	≤0.001	0.07	0.426	0.06	0.536
d550	Eating	−0.44	≤0.001	0.17	0.060	0.05	0.597
d640	Doing housework	−0.52	≤0.001	0.21	0.018	0.05	0.618
d570	Looking after one’s health	−0.30	0.001	0.10	0.272	0.01	0.881
d240	Handling stress and other psychological demands	−0.02	0.871	0.02	0.790	0.37	≤0.001
d230	Carrying out daily routine	−0.27	0.002	0.13	0.169	0.03	0.768
d770	Intimate relationships	−0.04	0.665	−0.11	0.265	0.19	0.058
d470	Using transportation	−0.27	0.003	0.17	0.063	0.18	0.053
d660	Assisting others	−0.18	0.049	0.02	0.823	0.06	0.482
d710	Basic interpersonal interactions	−0.17	0.069	0.02	0.854	0.16	0.079
d850	Remunerative employment	−0.08	0.406	−0.17	0.070	0.36	≤0.001
d920	Recreation and leisure	−0.10	0.278	−0.04	0.676	0.37	≤0.001

MBI: Modified Barthel Index, ECOG: Eastern Cooperative Oncology Group instrument, HADS: Hospital Anxiety and Depression Scale, r: the Spearman correlation coefficient.

Regarding the HADS, two categories in the Body Functions dimension and five in the Activity and Participation dimension showed significant positive correlations with its total score. Specifically, the HADS-Anxiety subscale correlated positively with 12 ICF-RS categories (2 in Body Functions, 10 in Activity and Participation), while the HADS-Depression subscale correlated with 5 categories (1 in Body Functions, 4 in Activity and Participation) (Table 6 and S4 Table).

In addition, the ECOG scores exhibited significant positive correlations with one category in the Body Functions dimension and two in the Activity and Participation dimension (Table 6).

## Discussion

The results demonstrate that the ICF-RS exhibits good reliability and evidence of criterion validity across several domains in Chinese cancer patients, although some validity measures were inconclusive. The scale effectively captures the multifaceted impacts of tumors on patients’ functioning and well-being, aligning closely with their daily activity levels and emotional status. High measurement consistency was observed, with robust internal consistency (overall Cronbach’s α and category-level α > 0.89), as well as substantial test-retest and inter-rater reliability. Construct validity and significant correlations with established health-related quality-of-life assessments further support the validity of the ICF-RS. These findings underscore its potential as a valuable tool in clinical practice and research, enabling comprehensive functional assessment and supporting individualized rehabilitation planning.

Regarding test-retest reliability, the majority of ICF-RS categories demonstrated high temporal stability, with Intraclass Correlation Coefficient (ICC) values exceeding 0.75, and several surpassing 0.90 (e.g., b152 Emotional functions, ICC = 0.99; d770 Intimate relationships, ICC = 1.00) as shown in the reliability analysis table. However, the b130 Energy and drive functions category yielded a relatively lower ICC of 0.77 (95% CI: 0.67–0.85). According to established methodological benchmarks (ICC 0.75–0.90 = “good”; > 0.90 = “excellent”) [50], these results indicate robust consistency. Notably, the b130 Energy and drive functions category yielded a relatively lower ICC of 0.77 (95% CI: 0.67–0.85). While still classified as “good,” this value diverges from a previous Polish validation study which reported “excellent” reliability for b130 (ICC = 0.982) over a 3-day interval in patients with neurological and musculoskeletal conditions [25]. This discrepancy may be explained by the distinct clinical trajectories of the two populations. Unlike the relatively stable functional status of patients with stroke sequelae or cerebral palsy, oncology patients often experience rapid fluctuations in vitality and physical fitness due to acute treatment side effects or disease progression. Furthermore, the interplay of psychological distress and environmental shifts in the early stages of cancer care may further influence retest scores, accounting for the moderated reliability observed in this specific category within the oncological rehabilitation context.

Similarly, in inter-rater reliability testing, most categories generated ICCs above 0.75, though *b455* Exercise tolerance, *b710* Joint mobility, *d770* Intimate relationships, and *d710* Basic interpersonal interactions fell below this threshold. The variability in *b455* and *b710* may reflect fluctuations in patients’ health status and treatment phase. This is consistent with prior research indicating that these physical domains exhibit lower inter-rater agreement even when structured rating guides are utilized. For instance, a Japanese study involving the ICF Generic-30 Set reported weighted κ values of 0.60 for *b455* and 0.65 for *b710*, marking them among the least consistent body-function categories studied [57]. In contrast, the lower agreement on *d770* and *d710* likely stems from the inherent subjective nature of psychosocial judgments, which are often susceptible to raters’ clinical experience. Quantitative evidence supports this: one study converting ADL assessments into ICF ratings found *d710* reached only κ = 0.44 (fair–moderate), substantially lower than the κ > 0.80 seen in self-care and mobility categories [58]. Likewise, *d770* has exhibited extremely poor agreement in other clinical populations, with a reported weighted κ of –0.04 in patients with rheumatoid arthritis, indicating near-zero inter-rater consistency [59]. Notably, higher inter-rater reliability for d770 has only been reported in long-term care settings, suggesting that prolonged observation of a subject may be a prerequisite for accurately assessing intimate interpersonal dynamics [60]. Furthermore, cultural nuances may have modulated these findings. An emphasis on familial privacy in certain contexts may limit a patient’s openness regarding intimate relationships (*d770*), while communication norms favoring emotional reserve and implicit understanding may complicate the objective rating of interpersonal interactions (*d710*) [61]. Given that achieving high inter-rater agreement on subjective items remains a recognized challenge [62], future research should explicitly investigate the impact of cultural factors on functional communication assessments. To enhance consistency, we recommend the development of standardized operational guidelines featuring behavioral anchors, specialized training in culturally sensitive communication, and the implementation of multi-rater consensus mechanisms.

The ICF-RS demonstrated strong construct validity in assessing tumor patients, with its body functions and activity/participation domains well-aligned with the primary concerns of this population. In the body functions dimension, low factor loadings for *b130* Energy and drive functions and *b152* Emotional functions may reflect the sample characteristics, which primarily included older patients in early disease stages. For such individuals, the impact of illness on core functions may not yet be fully manifested, suggesting that the ICF-RS may possess higher sensitivity in patients with more pronounced functional changes, such as those in middle-late stages or undergoing active treatment. A recent reference also indicated limited sensitivity of the ICF-RS in early-stage cancer patients, supporting this interpretation. Future research should include more patients from advanced disease stages to further validate the scale’s construct validity and clarify its optimal target population. Additionally, the expression of energy and emotional functions is influenced by patients’ overall medical condition, treatment stage, and available support systems. Although physical limitations and emotional distress are often inter-related in tumor patients [63,64], those in the early stages may not yet have experienced significant emotional impact [65]. It is hypothesized that when patients first confront constraints in physical functions or social participation, their immediate focus on adapting to these tangible challenges may cause their perception of energy and emotional well-being to take a back seat, which would consequently suppress the factor loadings of these categories. Nonetheless, it is also recognized that a patient’s emotional state can be affected by disease- and treatment-related stressors, which may have further contributed to the observed low factor loadings.

In the activity and participation dimension, low factor loadings were observed for *d240* Handling stress and other psychological demands, *d770* Intimate relationships, *d850* Remunerative employment, and *d920* Recreation and leisure. This may reflect the psychological and emotional stress experienced during treatment and recovery, which can impair the ability to handle psychological demands. However, as participants were in initial treatment stages, their emotional status may not yet have substantially affected activity and participation. Furthermore, their status as inpatients likely limited engagement in social, occupational, and recreational activities [66], constraining the expression of function in these areas. Overall health status and treatment-related impacts may have also contributed to the observed low factor loadings. Removing categories with loadings below 0.4 significantly increased the cumulative variance explained, suggesting that such items could be considered for deletion in future applications of the scale to tumor patients.

In the final version of construct validity, some items with low factor loading were deleted to optimize the scale’s structural validity. From a statistical perspective, removing items with low factor loadings improved the model fit and cumulative variance explained. However, from a clinical standpoint, this refinement involves a trade-off between statistical parsimony and diagnostic comprehensiveness. For instance, some excluded items might capture nuanced functional deficits that, while less frequent in the overall sample, remain critical for individualized rehabilitation planning in specific tumor subtypes. Nevertheless, this optimization approach is supported by similar methodological findings in other rehabilitation contexts. For example, a Rasch analysis of the ICF Core Set for low back pain demonstrated that collapsing rating categories and removing misfitting items resulted in a remaining set that better met the requirements of a unidimensional model, thereby enhancing internal construct validity without fundamentally altering the core functional dimensions the scale intended to measure [67]. Consequently, while the refined ICF-RS offers higher efficiency for rapid screening, clinicians should remain selective in utilizing the full set when a more granular assessment of environmental barriers or complex participation restrictions is required for long-term survivorship care.

The ICF-RS scores correlated strongly with other tumor-related assessment tools. Nearly 70% of ICF-RS categories correlated significantly with MBI scores, indicating effective measurement of daily living abilities. Emotion-related categories also showed significant correlations with the HADS and its subscales. However, only *b710* Mobility of joint functions, *d465* Moving around using equipment, and *d640* Doing housework correlated significantly with ECOG scores. This may be because the ECOG focuses on overall functional status and basic daily activities [45], making it less sensitive to more complex ICF-RS categories. The three correlated categories relate directly to physical capability and task performance, aligning better with ECOG’s measurement objectives. Furthermore, the relatively lower correlation between the ICF-RS and ECOG likely stems from a pronounced ceiling effect within the cohort [68]. While the ECOG is a robust predictor in advanced cancer, its discriminative power diminishes in rehabilitation or high-functioning populations where scores often cluster at 0–1. As a five-point categorical scale, the ECOG lacks the granularity to capture the nuanced functional variations that the multidimensional ICF-RS identifies. Consequently, this restricted variance in ECOG ratings ‘compresses’ its correlation with more continuous functional measures, highlighting the incremental value of the ICF-RS in detecting subtle clinical changes that global metrics overlook. Despite this, the positive correlations with MBI and specific ECOG categories reinforce the criterion-related validity of the ICF-RS and support its practical relevance in clinical settings.

Comparing the Chinese version of the ICF-RS with cancer-specific ICF-based tools, such as the Breast Cancer Core Set – Brief Chinese (BCSQ-BC) and cancer Functional Assessment Set (cFAS), reveals distinct advantages in terms of framework comprehensiveness, cross-condition comparability, and alignment with rehabilitation outcomes. While the cFAS primarily focuses on physical functions [69] and the BCSQ-BC is tailored specifically to the symptomatic profile of breast cancer [70], the ICF-RS is uniquely rooted in the WHO’s holistic bio-psycho-social model. This allows for a more systematic mapping of the Functioning–Participation–Environment spectrum within a single instrument. The findings regarding construct validity support the multidimensionality. Specifically, the Body Functions and Activities & Participation components explained 57.3% and 44.6% of the cumulative variance, respectively. This demonstrates that the ICF-RS captures a broader functional profile than the more narrowly focused cFAS. Furthermore, the ICF-RS utilizes a unified qualifier system that extends beyond basic activities of daily living, which is essential for comprehensive oncology care. This is evidenced by the criterion validity results: while 66.7% of the ICF-RS categories correlated significantly with the MBI, we also observed moderate correlations (ρ = 0.37–0.41, p < 0.001) between emotional function categories and the HADS. This capacity to integrate psychological demands and social participation—areas where the cFAS is relatively limited—makes the ICF-RS particularly suitable for multi-disciplinary goal-setting. While specialized tools like the BCSQ-BC provide high granularity for specific populations, the ICF-RS’s strong internal consistency (α = 0.87) and reliable performance underscore its superior utility as a universal functional language in broader clinical oncology settings.

Collectively, these results support the theoretical framework of the ICF and demonstrate the ability of the ICF-RS to capture functional complexity in a specific population. Specifically, the application of the Chinese ICF-RS provides clinicians with a standardized language to systematically identify multidimensional functional deficits, enabling the development of highly tailored rehabilitation interventions. Conversely, the absence of such a comprehensive tool may lead to an over-reliance on purely biological indicators, potentially overlooking critical environmental and social participation factors that are essential for a patient’s holistic recovery. Several limitations should be noted. First, all participants were in the initial stages of illness; future studies should include patients across various tumor progression stages. Second, assessments may have been influenced by social desirability bias, leading to over- or under-reporting of functional status. Third, while the three-day interval used for test-retest reliability was intended to minimize clinical fluctuations in the patients’ condition, it may have introduced recall bias, where participants’ familiarity with their previous responses could have influenced the assessment’s stability measures. Future research should aim to expand the utility of the Chinese ICF-RS in several key directions. First, while the current study established the cross-sectional validity of the scale, longitudinal studies are required to evaluate its responsiveness to clinical interventions and its sensitivity to functional changes over the course of cancer treatment. Second, as oncology rehabilitation increasingly moves towards digital health, future insights could be gained by integrating the ICF-RS into electronic health records (EHRs) or mobile health platforms, allowing for real-time functional monitoring and patient-reported data collection. Furthermore, conducting cross-cultural comparisons with other international versions of the ICF-RS could help standardize global functional outcomes in oncology, facilitating larger meta-analyses and the development of international clinical guidelines.

## Conclusions

The ICF-RS demonstrated robust structural validity and acceptable reliability in our tumor patient sample, indicating that the scale reliably captures the intended construct. However, the criterion validity was suboptimal, potentially due to differences among the criterion measures, sample heterogeneity, or cultural adaptation issues. Collectively, these findings support the ICF-RS as a promising instrument for functional assessment in tumor populations, while also underscoring the need for further validation.

Clinically, the findings highlight the practical value of applying the ICF framework and standardized tools in rehabilitation and nursing practice. With additional validation—particularly against established external criteria and in larger, more diverse samples—the ICF-RS could facilitate more comprehensive evaluations of functional status in tumor patients, support individualized treatment planning, and ultimately contribute to improved rehabilitation outcomes and quality of life. Future studies should focus on examining its criterion performance, cross-cultural equivalence, and the impact of routine clinical use on decision-making and patient outcomes.

## Supporting information

S1 Table The ICF Rehabilitation Set. (DOCX)

S2 Table The ICF-RS qualifiers used (n = 1055). Each category is evaluated using a 5-level scale (0–4), where higher levels indicate worse functional outcomes, along with their percentage (%).
(DOCX)

S3 Table The ceiling and floor effects for all 30 categories of the ICF-RS (n = 1055). (DOCX)

S4 Table Construct validity results for the original ICF-RS (n = 1055). KMO: Kaiser-Meyer-Olkin Measure of Sampling Adequacy.
(DOCX)

S5 Table Correlations between ICF-RS ratings and HADS scores (n = 1055). HADS-A: Hospital anxiety and depression scale-anxiety, HADS-D: Hospital anxiety and depression scale- depression,r: the Pearson correlation coefficient.
(DOCX)
