# Supplementary material for: The reliability and validity of rehabilitation set of the international classification of functioning, disability, and health in assessing Chinese tumor patients
Source: PLoS One. 2026 Jun 3;21(6):e0349504. doi: 10.1371/journal.pone.0349504 (PMC13232837; doi:10.1371/journal.pone.0349504)
Supplement: S1 Table — (DOCX) [file pone.0349504.s001.docx]

**S1 Table. The ICF Rehabilitation Set**

| **ICF Rehabilitation Set** | |
| --- | --- |
| b130 | Energy and drive functions |
| b134 | Sleep functions |
| b152 | Emotional functions |
| b280 | Sensation of pain |
| b455 | Exercise tolerance functions |
| b620 | Urination functions |
| b640 | Sexual functions |
| b710 | Mobility of joint functions |
| b730 | Muscle power functions |
| d230 | Carrying out daily routine |
| d240 | Handling stress and other psychological demands |
| d410 | Changing basic body position |
| d415 | Maintaining a body position |
| d420 | Transferring oneself |
| d450 | Walking |
| d455 | Moving around |
| d465 | Moving around using equipment |
| d470 | Using transportation |
| d510 | Washing oneself |
| d520 | Caring for body parts |
| d530 | Toileting |
| d540 | Dressing |
| d550 | Eating |
| d570 | Looking after one’s health |
| d640 | Doing housework |
| d660 | Assisting others |
| d710 | Basic interpersonal interactions |
| d770 | Intimate relationships |
| d850 | Remunerative employment |
| d920 | Recreation and leisure |

<https://std.samr.gov.cn/gb/search/gbDetailed?id=EB58F4DA9007B2A2E05397BE0A0A7D33>
