# Supplementary material for: The reliability and validity of rehabilitation set of the international classification of functioning, disability, and health in assessing Chinese tumor patients
Source: PLoS One. 2026 Jun 3;21(6):e0349504. doi: 10.1371/journal.pone.0349504 (PMC13232837; doi:10.1371/journal.pone.0349504)
Supplement: S2 Table — Each category is evaluated using a 5-level scale (0–4), where higher levels indicate worse functional outcomes, along with their percentage (%). (DOCX) [file pone.0349504.s002.docx]

**S2 Table. The ICF-RS qualifiers used (n=1055)**

|  | **ICF-RS category** | **Qualifiers (%)** | | | | |
| --- | --- | --- | --- | --- | --- | --- |
|  |  | **0** | **1** | **2** | **3** | **4** |
| **Body function** | b130 Energy and drive functions | 23(2.18) | 569(53.98) | 284(26.94) | 153(14.52) | 25(2.37) |
|  | b134 Sleep functions | 320(30.33) | 331(31.37) | 261(24.74) | 141(13.36) | 2(0.19) |
|  | b152 Emotional functions | 383(36.37) | 480(45.58) | 171(16.24) | 19(1.80) | 0(0.00) |
|  | b280 Sensation of pain | 510(48.43) | 254(24.12) | 191(18.14) | 97(9.21) | 1(0.09) |
|  | b455 Exercise tolerance functions | 825(78.35) | 122(11.59) | 67(6.36) | 21(1.99) | 18(1.71) |
|  | b620 Urination functions | 22(2.47) | 70(7.85) | 146(16.37) | 156(17.49) | 498(55.83) |
|  | b640 Sexual functions | 17(1.61) | 216(20.47) | 794(75.26) | 18(1.71) | 10(0.95) |
|  | b710 Mobility of joint functions | 911(86.35) | 128(12.13) | 13(1.23) | 3(0.28) | 0(0.00) |
|  | b730 Muscle power functions | 717(67.96) | 169(16.02) | 96(9.10) | 38(3.60) | 35(3.32) |
| **Activity**  **and Participation** | d240 Handling stress and other psychological demands | 830(78.67) | 91(8.63) | 87(8.25) | 40(3.79) | 7(0.66) |
|  | d410 Changing basic body position | 858(81.33) | 96(9.10) | 88(8.34) | 4(0.38) | 9(0.85) |
|  | d415 Maintaining a body position | 909(86.16) | 110(10.43) | 15(1.42) | 11(1.04) | 10(0.95) |
|  | d420 Transferring oneself | 918(87.01) | 100(9.48) | 19(1.80) | 6(0.57) | 12(1.14) |
|  | d450 Walking | 664(62.94) | 165(15.64) | 77(7.30) | 113(10.71) | 36(3.41) |
|  | d455 Moving around | 273(25.88) | 171(16.21) | 269(25.50) | 232(21.99) | 110(10.43) |
|  | d465 Moving around using equipment | 866(82.09) | 62(5.88) | 67(6.35) | 44(4.17) | 16(1.52) |
|  | d510 Washing oneself | 936(88.72) | 87(8.25) | 14(1.33) | 10(0.95) | 8(0.76) |
|  | d520 Caring for body parts | 967(91.66) | 46(4.36) | 22(2.09) | 10(0.95) | 10(0.95) |
|  | d530 Toileting | 966(91.56) | 51(4.83) | 17(1.61) | 18(1.71) | 3(0.28) |
|  | d540 Dressing | 853(80.85) | 181(17.16) | 7(0.66) | 6(0.57) | 8(0.76) |
|  | d550 Eating | 685(64.93) | 51(4.83) | 63(5.97) | 221(20.95) | 35(3.32) |
|  | d570 Looking after one’s health | 50(4.74) | 648(61.42) | 271(25.69) | 65(6.16) | 21(1.99) |
|  | d640 Doing housework | 8(0.76) | 835(79.30) | 179(17.00) | 30(2.85) | 1(0.09) |
|  | d230 Carrying out daily routine | 90(8.53) | 675(63.98) | 242(22.94) | 30(2.84) | 18(1.71) |
|  | d470 Using transportation | 136(13.93) | 798(81.76) | 40(4.10) | 0(0.00) | 2(0.20) |
|  | d660 Assisting others | 69(6.54) | 663(62.84) | 254(24.08) | 52(4.93) | 17(1.61) |
|  | d710 Basic interpersonal interactions | 5(0.47) | 345(32.70) | 469(44.45) | 215(20.38) | 21(1.99) |
|  | d770 Intimate relationships | 2(0.19) | 756(71.66) | 295(27.96) | 1(0.09) | 1(0.09) |
|  | d850 Remunerative employment | 336(31.94) | 88(8.37) | 211(20.06) | 189(17.97) | 228(21.67) |
|  | d920 Recreation and leisure | 55(5.21) | 419(39.72) | 351(33.27) | 174(16.49) | 56(5.31) |

Each category is evaluated using a 5-level scale (0-4), where higher levels indicate worse functional outcomes, along with their percentage (%).
