# Supplementary material for: The reliability and validity of rehabilitation set of the international classification of functioning, disability, and health in assessing Chinese tumor patients
Source: PLoS One. 2026 Jun 3;21(6):e0349504. doi: 10.1371/journal.pone.0349504 (PMC13232837; doi:10.1371/journal.pone.0349504)
Supplement: S3 Table — (DOCX) [file pone.0349504.s003.docx]

**S3 Table. The ceiling and floor effects for all 30 categories of the ICF-RS (n=1055)**

| **ICF-RS categories** | **Code** | **percentage of patients scoring 0 (%)** | **percentage of patients scoring 4 (%)** |
| --- | --- | --- | --- |
| Category 1 | b130 | 2.07 | 1.94 |
| Category 2 | b134 | 28.42 | 0.21 |
| Category 3 | b152 | 35.92 | 0.00 |
| Category 4 | b280 | 48.48 | 0.07 |
| Category 5 | b620 | 76.21 | 1.80 |
| Category 6 | b640 | 2.35 | 46.47 |
| Category 7 | b455 | 1.80 | 1.04 |
| Category 8 | b710 | 85.62 | 0.00 |
| Category 9 | b730 | 68.05 | 2.56 |
| Category 10 | d410 | 79.46 | 0.83 |
| Category 11 | d415 | 81.81 | 0.97 |
| Category 12 | d420 | 87.62 | 1.18 |
| Category 13 | d450 | 87.97 | 1.31 |
| Category 14 | d465 | 60.79 | 3.73 |
| Category 15 | d455 | 25.59 | 10.30 |
| Category 16 | d510 | 82.78 | 1.66 |
| Category 17 | d520 | 89.42 | 1.04 |
| Category 18 | d530 | 92.32 | 1.18 |
| Category 19 | d540 | 92.12 | 0.28 |
| Category 20 | d550 | 80.77 | 0.76 |
| Category 21 | d640 | 63.21 | 3.53 |
| Category 22 | d570 | 4.56 | 2.01 |
| Category 23 | d240 | 0.55 | 0.07 |
| Category 24 | d230 | 9.13 | 1.45 |
| Category 25 | d770 | 13.83 | 0.14 |
| Category 26 | d470 | 6.36 | 1.66 |
| Category 27 | d660 | 0.41 | 2.01 |
| Category 28 | d710 | 0.14 | 0.07 |
| Category 29 | d850 | 29.32 | 19.50 |
| Category 30 | d920 | 5.74 | 4.91 |
