# Supplementary material for: The reliability and validity of rehabilitation set of the international classification of functioning, disability, and health in assessing Chinese tumor patients
Source: PLoS One. 2026 Jun 3;21(6):e0349504. doi: 10.1371/journal.pone.0349504 (PMC13232837; doi:10.1371/journal.pone.0349504)
Supplement: S4 Table — KMO: Kaiser-Meyer-Olkin Measure of Sampling Adequacy. (DOCX) [file pone.0349504.s004.docx]

**S4 Table. Construct validity results for the original ICF-RS (n=1055)**

| **Component and category** | | | **Factor loading coefficient** | | **Common factor variance** |
| --- | --- | --- | --- | --- | --- |
|  |  |  | **Body functions** | **Activity**  **and Participation** |  |
| **Body functions** | b130 | Energy and drive functions | 0.20 | 0.33 | 0.15 |
|  | b134 | Sleep functions | **0.52** | 0.04 | 0.28 |
|  | b152 | Emotional functions | 0.38 | 0.10 | 0.15 |
|  | b280 | Sensation | **0.55** | 0.21 | 0.34 |
|  | b620 | Urination functions | **0.50** | 0.00 | 0.25 |
|  | b640 | Sexual functions | **0.41** | 0.03 | 0.17 |
|  | b455 | Exercise tolerance functions | **0.46** | 0.32 | 0.31 |
|  | b710 | Mobility of joint functions | 0.33 | **0.44** | 0.30 |
|  | b730 | Muscle power functions | 0.13 | **0.56** | 0.34 |
| **Activity**  **and Participation** | d410 | Changing basic body position | 0.36 | **0.64** | 0.54 |
|  | d415 | Maintaining a body position | 0.32 | **0.65** | 0.53 |
|  | d420 | Transferring oneself | 0.07 | **0.86** | 0.74 |
|  | d450 | Walking | 0.11 | **0.89** | 0.80 |
|  | d465 | Moving around using equipment | **0.62** | 0.34 | 0.50 |
|  | d455 | Moving around | **0.69** | 0.38 | 0.62 |
|  | d510 | Washing oneself | 0.23 | **0.73** | 0.59 |
|  | d520 | Caring for body parts | 0.10 | **0.89** | 0.80 |
|  | d530 | Toileting | 0.10 | **0.90** | 0.83 |
|  | d540 | Dressing | 0.08 | **0.90** | 0.81 |
|  | d550 | Eating | 0.24 | **0.72** | 0.57 |
|  | d640 | Doing housework | **0.67** | 0.38 | 0.59 |
|  | d570 | Looking after one’s health | **0.55** | **0.57** | 0.63 |
|  | d240 | Handling stress and other psychological demands | 0.06 | **0.40** | 0.16 |
|  | d230 | Carrying out daily routine | **0.52** | **0.53** | 0.56 |
|  | d770 | Intimate relationships | 0.36 | 0.00 | 0.13 |
|  | d470 | Using transportation | **0.51** | **0.55** | 0.56 |
|  | d660 | Assisting others | 0.33 | **0.41** | 0.27 |
|  | d710 | Basic interpersonal interactions | **0.55** | 0.11 | 0.31 |
|  | d850 | Remunerative employment | -0.05 | 0.07 | 0.01 |
|  | d920 | Recreation and leisure | 0.30 | 0.29 | 0.17 |
| Eigenvalue (after rotation)  Variance explained (after rotation)  Cumulative variance explained (after rotation)  KMO  Bartlett’s sphericity test value degrees of freedom *p* value | | | 8.34 | 4.65 | - |
|  |  |  | 27.79% | 15.50% | - |
|  |  |  | 27.79% | 43.29% | - |
|  |  |  | 0.94 | | |
|  |  |  | 14808 | | |
|  |  |  | 435 | | |
|  |  |  | ≤0.001 | | |

KMO: Kaiser-Meyer-Olkin Measure of Sampling Adequacy
