# Supplementary material for: The reliability and validity of rehabilitation set of the international classification of functioning, disability, and health in assessing Chinese tumor patients
Source: PLoS One. 2026 Jun 3;21(6):e0349504. doi: 10.1371/journal.pone.0349504 (PMC13232837; doi:10.1371/journal.pone.0349504)
Supplement: S5 Table — HADS-A: Hospital anxiety and depression scale-anxiety, HADS-D: Hospital anxiety and depression scale- depression,r: the Pearson correlation coefficient. (DOCX) [file pone.0349504.s005.docx]

**S5 Table. Correlations between ICF-RS ratings and HADS scores (n=1055)**

| **ICF-RS** | | **HADS-A** | | **HADS-D** | |
| --- | --- | --- | --- | --- | --- |
|  |  | **r** | ***p*** | **r** | ***p*** |
| **Body functions** | |  |  |  |  |
| b130 | Energy and drive functions | 0.20 | 0.027 | 0.10 | 0.290 |
| b134 | Sleep functions | 0.06 | 0.513 | 0.03 | 0.728 |
| b152 | Emotional functions | 0.37 | ≤0.001 | 0.37 | ≤0.001 |
| b280 | Sensation | 0.20 | 0.032 | 0.17 | 0.065 |
| b620 | Urination functions | -0.03 | 0.724 | -0.03 | 0.726 |
| b640 | Sexual functions | -0.03 | 0.807 | -0.04 | 0.719 |
| b455 | Exercise tolerance functions | 0.06 | 0.504 | -0.02 | 0.793 |
| b710 | Mobility of joint functions | -0.04 | 0.700 | -0.02 | 0.855 |
| b730 | Muscle power functions | 0.20 | 0.030 | 0.12 | 0.208 |
| **Activity and Participation** | |  |  |  |  |
| d410 | Changing basic body position | 0.00 | 0.980 | 0.04 | 0.671 |
| d415 | Maintaining a body position | 0.01 | 0.941 | 0.06 | 0.535 |
| d420 | Transferring oneself | 0.18 | 0.047 | 0.19 | 0.034 |
| d450 | Walking | 0.20 | 0.025 | 0.16 | 0.089 |
| d465 | Moving around using equipment | 0.13 | 0.165 | 0.16 | 0.088 |
| d455 | Moving around | 0.02 | 0.856 | 0.09 | 0.315 |
| d510 | Washing oneself | 0.16 | 0.073 | 0.01 | 0.885 |
| d520 | Caring for body parts | 0.15 | 0.095 | 0.02 | 0.836 |
| d530 | Toileting | 0.16 | 0.079 | 0.01 | 0.926 |
| d540 | Dressing | 0.08 | 0.380 | 0.03 | 0.711 |
| d550 | Eating | 0.09 | 0.330 | 0.02 | 0.865 |
| d640 | Doing housework | 0.12 | 0.178 | -0.02 | 0.861 |
| d570 | Looking after one’s health | 0.09 | 0.335 | -0.06 | 0.531 |
| d240 | Handling stress and other psychological demands | 0.45 | ≤0.001 | 0.23 | 0.012 |
| d230 | Carrying out daily routine | 0.06 | 0.489 | 0.00 | 0.979 |
| d770 | Intimate relationships | 0.23 | 0.018 | 0.07 | 0.475 |
| d470 | Using transportation | 0.24 | 0.010 | 0.12 | 0.193 |
| d660 | Assisting others | 0.01 | 0.877 | 0.12 | 0.201 |
| d710 | Basic interpersonal interactions | 0.18 | 0.047 | 0.10 | 0.279 |
| d850 | Remunerative employment | 0.35 | ≤0.001 | 0.29 | 0.001 |
| d920 | Recreation and leisure | 0.31 | ≤0.001 | 0.33 | ≤0.001 |

HADS-A: Hospital anxiety and depression scale-anxiety, HADS-D: Hospital anxiety and depression scale- depression,,r: the Pearson correlation coefficient.
